# Resident funding and care home quality: a retrospective observational analysis of the impact of the two-tier care system in England

**DOI:** 10.1093/ageing/afaf100

**Published:** 2025-05-03

**Authors:** Anders Bach-Mortensen, Benjamin Goodair, Michelle Degli Esposti, Christine Corlet Walker

**Affiliations:** Blavatnik School of Government, University of Oxford, Oxford, UK; Department of Social Sciences and Business, Roskilde University, Roskilde, Denmark; Institute for Firearm Injury Prevention, University of Michigan, Ann Arbor, MI, USA; Institute for Firearm Injury Prevention, University of Michigan, Ann Arbor, MI, USA; Centre for Environment and Sustainability, University of Surrey, Guildford, Surrey, UK

**Keywords:** outsourcing, two-tier care, care home funding, quality of care, older people

## Abstract

**Background:**

Adult social care in England operates in a two-tier system of self-funded and state-funded residents. It is unclear, however, whether resident funding source impacts care home quality.

**Methods:**

We conducted a nationwide retrospective observational analysis of care homes in England (*n* = 28 239 Provider Information Return entries for 14 444 care homes, representing ~367 653 residents), 2021–23, to examine the relationship between resident funding (self- or state-funded) and care home quality (inspection ratings by the industry regulator). We linked data from the Care Quality Commission’s Provider Information Return to inspection ratings, area deprivation, and care home and resident characteristics. We modelled a series of logistic regressions, incorporating interaction terms to investigate the interrelationships between ownership (for-profit, third sector, public) and area deprivation.

**Findings:**

Care homes with more self-funded residents were more likely to have better inspection ratings [odds ratio for each percentage of self-funded residents: 1.01, 95% confidence interval (CI) 1.008–1.012, *P* < .001]. The effect of self-funded residents on care quality was largest amongst for-profit homes and not statistically significant for third sector and public homes. For homes without self-funders, third sector and public providers were 14.0 (95% CI: 10.1–17.8, *P* < .001) and 6.9 (95% CI: 4.1–9.7, *P* < .001) percentage points more likely to be rated higher than for-profit homes.

**Conclusions:**

The quality of for-profit care homes is strongly influenced by the proportion of self-funded residents, whilst third and public sector homes provide consistent care regardless of resident funding source. This strongly impacts care equity for residents nationwide, as the concentration of self-funders is largely determined by area wealth.

## Key Points

- Adult social care in England operates on a two-tier system of self-funded and state-funded residents.
- Using novel and population level data, we find that self-funded residents are more likely to be in good quality provision.
- For-profit homes with a higher percentage of self-funders perform substantially better than those serving state-funded residents.
- The effect of self-funded residents on care quality was not statistically significant for third and public sector homes.

## Background

Adult social care services in England operates in a two-tier system, in which residential adult social care facilities serve a mix of (1) residents that pay, in part or in full, for their care services (self-funders) and (2) residents whose costs are covered by the state (state-funders). State-funding eligibility is determined by the assets and savings available to people in need of care [1]. Of the four UK nations, the state-funding criteria are the lowest and least generous in England, and access to state-funded care has worsened in recent years due to reduced funding from central government and increased care costs [2]. This means that there are more self-funded residents and that more social care recipients will, at some point in their care trajectory, be required to self-fund. The knock-on effects of increased self-funding on service quality and care equity is not well understood.

Historically, there has been an absence of data on the resident funding mix in terms of how many residents are state- and self-funded, what types of homes serve what residents and whether funding source is associated to quality of care. Recent data from the Office for National Statistics (ONS) have improved the documentation at national and Local Authority (LA) level. The ONS estimates that, from March 2022 to February 2023, 37% of all care home residents were self-funders, and that this percentage is increasing [3, 4]. It further shows that 1.3% of homes (representing 1.6% of beds) only serve self-funders, whereas 39% homes (but only representing 13.6% of beds) only had state-funders. Most homes (59.7%) serve a mix of self- and state-funded residents [3].

However, it is unclear how resident funding source influences the quality and accessibility of care services. This is a key policy and research gap, in that there are several ways that funding source may influence care provision. It is widely reported by the sector that inflation and increased care costs are pushing care homes to charge higher fees to self-funders to subsidise state-funded residents [5–7]. For example, the 2018 Competition and Markets Authority report found that self-funders pay 41% higher fees, on average, than state-funders in the same homes [8]. A discrepancy in care costs between state- and self-funded residents may directly impact care quality and accessibility in market-based provision. If the profitability of care homes depends on access to self-funded residents, it follows that profit-oriented homes will organise their services in order to (1) cater to self-funded residents and (2) focus on areas with a higher concentration of self-funded residents. Previous research has flagged that the two-tier system could lead to a resident selection effect amongst providers [5, 6], and the industry regulator, the Care Quality Commission (CQC), recently reported that, because state-funded places are less profitable, state-funded residents and residents in more deprived areas are at higher risks of receiving inadequate or poor quality care [9].

Although adult social care is technically a mixed market with both for-profit, third sector (non-profit) and local authority (public) provision, it has become almost entirely outsourced to the third and for-profit sector: in 2023, >85% of homes (representing 88.7% registered beds) were operated by for-profit providers [10]. When most provision is for-profit, it follows that profit-oriented incentives have the potential to have an aggregate and detrimental impact on the sector. The risk of providers adapting such that they can best attract lucrative self-funded residents is especially acute when state-funded resident fees are underfinanced [11, 12]. Existing work has found that care home competition is associated with reduced quality and lower costs, particularly in areas with a higher concentration of state-funded residents [13]. Yet, the link between funding source and care home quality, and how this has interacted with the outsourcing of social care, is poorly understood.

There is renewed urgency to evaluate the impact of the two-tier care system: In July 2024, the Government announced that it was cancelling the planned reform on changing how people pay for social care in England [14]. Amongst other initiatives, the reform proposed to change the self-funding asset threshold from £23 250 to £100 000, increase LA funding to enable higher state-funded resident fees, allow self-funders to access care for the same fees as state-funded residents and to introduce a cap (£86 000) on lifetime care costs [15]. These initiatives were meant to make more residents eligible for state-funded care, reduce the financial reliance on self-funded residents by paying higher state-funded fees, and to reduce the risk of individuals and families facing very large care costs. By abandoning this plan, more people will have to self-fund their care, without any state guarantee in terms of what these costs can accumulate to.

It has not previously been possible to examine the links between resident funding source, quality, area and ownership due to a lack of reliable data. Using novel care home–level data from the Provider Information Return (PIR) from the CQC, we investigate the relationship between resident funding source and care home quality and examine whether this relationship is moderated by care home ownership (for-profit, third sector, public) and area deprivation.

## Methods

We conducted a national observational analysis of all care homes in England from March 2021 to September 2023. The results are reported following the RECORD (REporting of studies Conducted using Observational Routinely collected health Data) guidelines [16].

### Data sources

Data were obtained via a Freedom of Information (FOI) request from the CQC’s PIR dataset covering March 2021–September 2023. The PIR is a mandatory annual survey, and all registered providers are required by law to fill it out. We received access to 30 months of this survey, which is submitted on a rolling basis, meaning we have at least two returns for each home except for those that opened or closed in that period. Care homes that do not submit the PIR in time will automatically receive a ‘Requires improvement’ or lower rating in the ‘Well-led’ inspection domain until it is submitted [17]. Prior to receiving, the data were linked via location ID and postcode by CQC data officers to two additional data sources. First, the FOI data were linked to the CQC registration data to identify care home ownership (for-profit, third sector and LA) and overall CQC inspection rating for each care home. Second, each care home was linked via postcode to Lower layer Super Output Areas [18]. The final cleaned dataset included 27 860 PIR entries (covering 14 444 unique care homes) with location level data. See pages 2–3 and Table A1 in the Appendix for details on our procedure for cleaning the dataset and information on missing observations.

### Exposure: resident funding source

The PIR includes detailed questions on funding source (Table 1) [19]. We defined funding source as the percentage of self- and state-funders relative to all residents.

**Table 1:** Funding source

Funding type	Question	Elaboration in PIR form
State-funders	‘How many of the people who use your service (i) are funded in full or in part by their local authority, or (ii) receive NHS Continuing Health Care?’ [19]	‘Include in your number those who pay user charges towards local authority funded care; those who pay using a local authority personal budget, or those who have someone paying a 3rd-party top-up on their behalf.’ [19]
Self-funders	‘How many other people use your service?’ [19]	‘This should include self-or charity-funded users and include those receiving NHS Funded Nursing Care, and also those paying the full cost through their local authority.’ [19]

Note: The form further specifies that the sum of self-funders and state-funders should equal the total number of residents [19].

### Outcome: care home quality

Our primary outcome was the latest overall CQC inspection rating specified in the linked data. We collapsed the 4-point CQC inspection rating scale into a binary variable of ‘Inadequate’/‘Requires improvement’ versus ‘Good’/‘Outstanding’. Findings were similar when analysing the original ordered 4-point rating using ordered logistic regression (see Appendix Table A2).

### Moderators: ownership and area deprivation

We also extracted information on care home ownership and area deprivation. Specifically, ownership was operationalised in three categories: for-profit (all private companies, individuals and partnerships without a charity number), third sector (registered charities) and public (local authority and National Health Service (NHS)) provision. We defined area deprivation using area level area deprivation deciles from the income Deprivation Affecting Older People Index (IDAOPI) [18], which ranks postcodes based on the percent of older people living in deprivation. In Table A8 in the Appendix, we replicate our results using the Index of Multiple Deprivation.

### Confounders: care home and resident characteristics

We also identified several hypothesised confounders based on home, resident and staff characteristics. These included number of residents, service user band registrations, whether the home includes nursing, residents’ care needs (proportion of residents with dementia, mental health needs, learning disabilities/autism and physical disabilities) and the full-time staff-to-resident ratio. See Appendix page 3 for a full list of control variables and Table A5 for a replication of the results using only resident characteristics.

### Statistical analysis

We ran a series of unadjusted and adjusted logistic and OLS regressions with care home quality (CQC inspection ratings) as the outcome. In our adjusted analyses, we control for care home registration (e.g. if the home is registered for older people or nursing), the number of residents, the number of employees, resident needs (mental health, dementia, physical and learning disabilities) and whether the care home closed during the study period, and we add year fixed effects. All analyses were conducted in R (v4.4.1) and Stata (v17.0).

First, we ran logistic regressions with fixed effects for the PIR submission year. To include all observations (and not just the latest PIR submission), we clustered the standard errors at care home level to account for repeat observations. The results are similar when only including the latest PIR observations (Table A4, Appendix).

Second, to investigate whether the effect of funding source varied by ownership, area deprivation and resident needs, we conducted several interaction models, allowing the associations between resident funding source and quality to vary by these variables [20] (see Figures A3–A7 in the appendix for all interaction models). We visualised moderation analyses using marginal effects and predicted probabilities, which are more robust to model specification than odds ratios (ORs) and better suited to capture and interpret non-linear effects [21].

## Results

The final dataset includes 28 880 observations over 3 years (2021–23), representing 14 444 unique care homes (13 260 active and 1184 closed locations) and 367 653 residents of which 35.8% (131 502) of individuals are reported as self-funders.

For-profit homes had the highest average percentage of self-funders (27.1%), followed by third sector (23.5%) and LA (10.2%) provision (Table 2). The average proportion of self-funded residents was higher in care homes located in less deprived areas: in the least deprived areas (IDAOPI = 10), care homes had an average of 39.76% self-funded residents, whereas in the most deprived areas (IDAOPI = 1), care homes had an average of only 11.51% self-funded residents. Provider size also influenced the composition of resident funding. Smaller homes—defined by fewer residents and staff—had the smallest percentage of self-funders, whereas larger homes had the highest (see Table 2). Homes registered for older residents and with nursing care had an average of 34.5% and 33.3% self-funders, whilst care homes not registered for older people had very few self-funders (2.64%). The number and percentage of state- and self-funded residents for each care home category can be found in Table A9 in the Appendix.

**Table 2:** Proportion of self- and state-funded residents in active care homes (September 2023)

	Self-funded residents (average care home %)	State-funded residents (average care home %)	Total care homes (*n*)
Care home ownership (*N* = 13 256)			
For-profit	27.08	72.92	11 306
Local Authority (public)	10.20	89.80	323
Third sector (non-profit)	23.52	76.48	1627
Area deprivation [IDAOPI deciles (*N* = 13 241)]			
1 (most deprived)	11.51	88.49	831
2	15.99	84.01	1203
3	19.04	80.96	1431
4	21.90	78.10	1493
5	25.39	74.61	1633
6	28.36	71.64	1583
7	29.88	70.12	1458
8	33.23	66.77	1355
9	35.24	64.76	1290
10 (least deprived)	39.76	60.24	967
Overall ratings (*N* = 13 128)			
Inadequate	23.46	76.54	200
Requires improvement	23.42	76.58	2348
Good	26.36	73.64	10 011
Outstanding	36.58	63.42	571
Number of residents (*N* = 13 155)			
1–9	3.14	96.86	3723
10–19	25.14	74.86	2359
20–49	37.80	62.20	5398
>50	40.95	59.05	1677
Number of staff (*N* = 13 155)			
1–9	6.28	93.62	1357
10–19	14.65	85.35	3245
20–49	31.32	68.68	5771
>50	38.66	61.24	2766
Location status (*N* = 14 444)			
Active	26.23	73.77	13 260
Closed	27.59	72.31	1184
Care home registrations			
Older residents (*N* = 13 256)			
Older residents	34.53	65.47	9806
No older residents	2.64	97.36	3450
Nursing (*N* = 13 236)			
With nursing	33.27	66.73	3815
Without nursing	23.42	76.58	9419
Residents’ care needs			
One or more residents with dementia (*N* = 13 256)			
No	3.11	96.89	4189
Yes	36.92	63.08	9065
One or more residents with a physical disability (*N* = 13 256)			
No	18.37	81.63	4038
Yes	29.68	70.32	9218
One or more residents with mental health needs (*N* = 13 256)			
No	26.30	73.70	12 194
Yes	25.46	74.54	1062
One or more residents with a learning disability or autism (*N* = 13 256)			
No	43.17	56.83	5824
Yes	12.95	87.05	7432

This table shows the latest observation from all active providers as of September 2023. The *N*s vary slightly for each category due to missing information on area deprivation and inspection ratings for some care homes. The percentages are calculated at care home level, and should not be read as the percentage of the number of self- and state-funded residents across categories (this can be found in Table A9 in the Appendix). Note that the residents’ care need categories are not mutually exclusive and may overlap.

**Figure 1:**
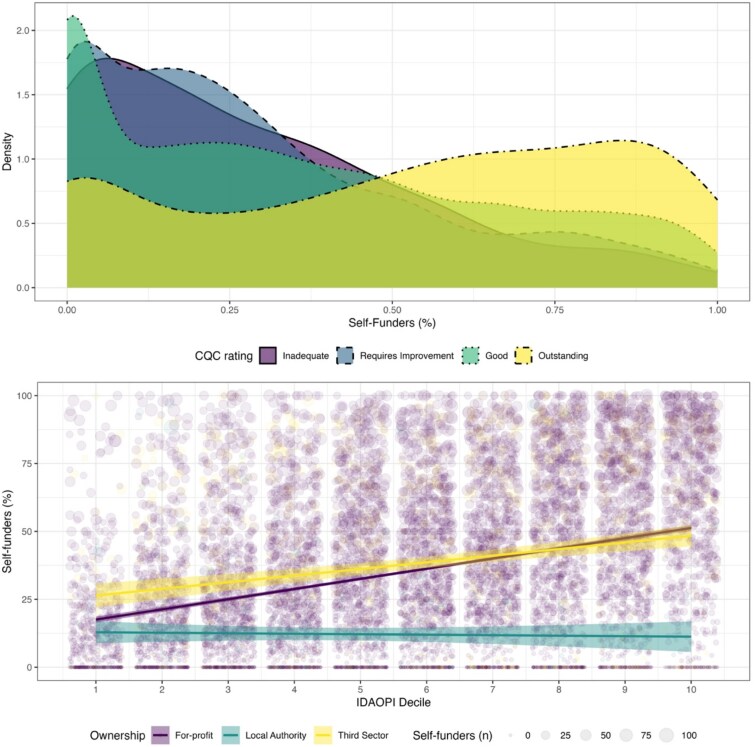
Density and bubble plots for homes with older people. Note: These numbers are estimated at care home (and not resident) level. In the bottom panel, each bubble represents a care home, which each have uniform opacity and shading. Darker areas represent overlapping care home bubbles.

Figure 1 shows the care home density and distribution spread of overall ratings and IDAOPI deciles, for homes registered to serve older residents. The density of outstanding ratings increases with self-funding percentage, whereas the distribution of self-funded residents for the other ratings is comparable. It further shows that care homes with the highest proportion of self-funded residents are clustered in less deprived areas, and that the association between the number of self-funders and deprivation deciles is strongest amongst for-profit homes. The percentage of self-funding residents in homes not registered for older residents is not illustrated below, as very few residents in these homes are self-funded (see appendix Figure A1).

Table 3 shows the adjusted and unadjusted associations between quality and resident funding source. There was a statistically significant association between resident funding source and care home quality, where a greater percentage of self-funders increased the likelihood of homes being rated of higher quality, irrespective of adjusting for covariates. Using the full sample of care homes, one percentage point increase in self-funded residents is associated with a 1.01 higher likelihood of a better rating—suggesting that homes with 20% self-funders are 1.2 times more likely to be rated higher than homes that only serve state-funded residents. This association is larger (OR: 1.012; *P* < .001) amongst homes registered for residents >65 years old (column 4, Table 3). When running separate regressions for each ownership category, the link between self-funders and quality was only statistically significant for for-profit homes (Table A7, Appendix).

**Table 3:** Associations between resident funding source and care home quality

	Unadjusted ORs [95% CIs]		Adjusted ORs [95% CIs]	
	[1]	[2]	[3]	[4]
Self-funders (%)	1.003^***^ [1.002, 1.005]	1.002^***^ [1.001, 1.004]	1.010^***^ [1.008, 1.012]	1.012^***^ [1.010, 1.014]
Care home ownership (*reference: for-profit*)				
Local Authority (public sector)	2.170^***^ [1.537, 3.064]		2.182^***^ [1.535, 3.102]	2.012^***^ [1.374, 2.946]
Third sector (non-profit)	1.556^***^ [1.349, 1.794]		1.348^***^ [1.161, 1.563]	1.181 [0.983, 1.420]
Area deprivation (IDAOPI deciles)		1.021^*^ [1.004, 1.038]	1.008 [0.991, 1.026]	1.006 [0.986, 1.027]
*N*	28 316	28 295	27 860	20 599
Unique location clusters	14 300	14 287	14 177	10 532
Fixed effects year	Yes	Yes	Yes	Yes
Care home registrations	All	All	All	Older residents
Control variables	No	No	Yes	Yes

See Table A3 in the Appendix for full regression results. Note: IDAOPI = 1 most deprived areas; 10 = least deprived areas. The outcome is the overall CQC inspection rating (‘Inadequate’/‘Requires improvement’ vs ‘Good’/‘Outstanding’). All effects are in odds ratios; 95% confidence intervals in brackets. The *N*s in the adjusted models are slightly lower than in the unadjusted models due to missing observations on some of the control variables. Specifically, model 3 has 110 fewer care homes (comprising 435 observations) than in model 2 due to missing values in the staff–resident ratio variable. The *N* in model 4 is lower than the other models because it only includes care homes registered for older people. See pages 2–3 in the Appendix for details on our procedure for removing observations and information on missing observations. ^*^P < .05, ^***^P < .001.

Public and third sector ownership was associated (*P* < .001) with higher quality in all model specifications: LA and third sector homes are 2.18 and 1.35 times more likely to be rated Good or Outstanding compared to for-profit provision, controlling for covariates. The percentage of dementia residents is associated with a smaller likelihood (*P* < .001) of a Good/Outstanding rating. Area deprivation (IDAOPI) was not associated with inspection ratings in the fully adjusted models (see Appendix Table A3 for the full regression results). However, the interaction results suggest that the effect of self-funded residents on quality is higher in less deprived areas (Table A6 and Figure A2 in the Appendix).

**Figure 2:**
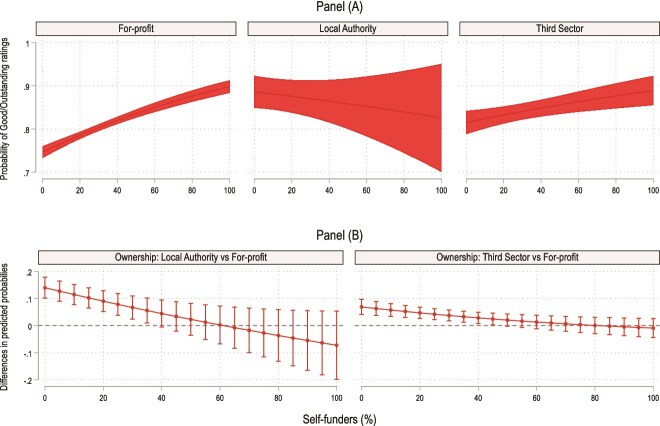
Predicted probabilities to be rated Good or Outstanding by care home ownership. The marginal effects models are calculated with fixed effects for reporting year, and all standard errors are clustered at the location level. The models adjust for the same variables as in model 3 in Table 2. See Appendix Table A6 for the full interaction models.

Figure 2 visualises the interaction effect of ownership on the association between resident funding source and quality (panel A) and the differences in predicted probabilities between ownership for different levels of self-funders (panel B) (see Appendix Table A6 for the full interaction models).

The figure shows that for-profit and third sector homes with a higher proportion of self-funded residents are more likely to be rated of higher quality, whereas the quality of LA homes is consistent regardless of resident funding source (panel A). The association between percentage of self-funded residents and quality is strongest amongst for-profit homes. For example, the predicted probability for a for-profit home with 20% versus 80% self-funded residents to have a ‘Good’/‘Outstanding’ overall rating is 78.1% and 87.3% (9.2%-point difference), respectively. In comparison, the predicted probabilities for a third sector (non-profit) home with the 20% versus 80% self-funded residents are 85.6% and 87.9% (2.3%-point difference). The differences in predicted probabilities between ownership attenuate with the level of self-funders (panel B).

The difference between for-profit and third sector/public provision is largest for homes with no self-funders: LA and third sector homes that only serve state-funded residents are 14.0 and 6.9% points (*P* < .001) more likely to be rated Good/Outstanding compared to for-profit homes. Ownership differences in quality were generally not statistically significant for homes with >50% self-funded residents—which represents around 22% of care homes and 30% of the residents in England.

## Discussion

This paper uses nationwide care home data to estimate the impact of the two-tier system on care quality. Our results highlight disparities in the quality of care provided to self-funded and state-funded residents in residential adult care in England. The analysis shows that homes with more self-funded residents are more likely to be rated higher by the English industry regulator, the CQC. This association is strongest and only statistically significant amongst private for-profit care homes.

The findings corroborate past research that the quality of for-profit care homes is rated worse, on average, than in public and third sector provision [22–24], but adds to the existing research in several ways. Past research on care home ownership has not been able to empirically account for resident characteristics and has therefore been unable to test whether variation in quality is driven by resident needs and funding source. This analysis finds that public and third sector provision receive better ratings, even when adjusting for funding source and resident characteristics. Furthermore, we find that the impact of funding source on quality varies by ownership, and that homes that serve only, or a majority of, state-funded residents are much more likely to receive higher quality services in third sector and public provision.

The relationship between self-funding and quality in for-profit homes is not surprising at face value. Self-funded residents typically pay substantially higher fees than state-funded residents [5, 8], providing homes with more self-funded residents more resources on average. Additionally, self-funders often have greater agency to choose higher quality homes, potentially creating a selection effect. However, despite the growing self-funding population, most residents (63%) remain state-funded [25], and only a small minority of homes (1.3%) exclusively serve self-funded residents. This suggests that state-funded residents remain important to many providers. These placements may be considered ‘safer’ and can ensure good occupancy levels for homes with mixed funding streams [8, 26]. Furthermore, block contracts or inclusion on local authority provider lists can contribute to a home’s sustainability.

These findings are significant for the sector. We estimate that 24.6% of residents in currently active homes in the most deprived areas live in poorer (inadequate/requires improvement) provision, whereas >87% of self-funded residents in the least deprived postcodes are in Good/Outstanding-rated care homes. If care quality is determined by the concentration of self-funded residents, this will negatively impact quality for residents in deprived areas and in provision that primarily houses state-funded residents. Previous research has found that competition leads to lower inspection ratings and costs in less prosperous areas [13, 27]. These results suggest that the effect of competition appears to influence for-profit and non-profit (third sector and LA) homes differently. When examining future reform of the sector, ownership therefore should not be disregarded, as it appears to fundamentally influence the quality and accessibility of care services.

## Limitations and further research

A key caveat of our analysis is endogeneity (i.e. reverse causality), as self-funded residents might have more agency than state-funded residents to self-select into higher quality homes. As such, providers might alter their quality to attract more self-funding residents, rather than self-funders having a direct impact on quality. Additionally, the level of competition each care home faces likely plays a significant role in determining quality levels, and this may not be fully captured in our analysis. The stability of self-funding rates and competitive environments year-over-year for individual care homes may also impact our ability to isolate these effects. Furthermore, because the data are anonymised, we cannot account for variability in the consistency of inspections across the four CQC regions. Finally, CQC measures of quality may not fully reflect residents’ experiences of quality.

Future research could further this work by analysing more granular resident-level data, linking individual care needs and funding sources. Furthermore, obtaining the actual fees charged to self-funded versus state-supported residents would enable analysis of the role of cost discrepancies on quality. Care quality is more than inspection ratings, and more research on the experiences of residents and families on navigating the two-tier system is needed [7], especially on the process of transitioning from self- to state-funded care. Lastly, our findings may not generalise to countries with different regulatory contexts. As more countries are turning to similar care models [28], replicating this analysis in other countries would improve our understanding of the impact of a two-tier funding model on care quality and equity.

## Conclusion

Decades of insufficient social care funding has created a two-tier social care system in which state-funded care is harder to access and where providers need to attract self-funded residents to cover costs. Because fewer residents are eligible for state-funded care, social care providers have been afforded the market power to charge self-funders more. In this paper, we show that coupling a two-tier care system with marketised provision interacts in ways that exacerbate geographical disparity. The mixed market system was designed to deliver higher quality care services for *all* residents at lower costs. Our findings suggest that outsourced provision strongly favours self-funded residents, but primarily in for-profit homes. These inequalities in care access and quality are likely to be exacerbated going forward. Scrapping and further delaying reform on social care funding commits more people to continue having to self-fund their care, for providers to continue to rely on current levels of funding and for state-funded residents to navigate an increasingly inaccessible care system.

## Supplementary Material

aa-24-1752-File002_afaf100

## Data Availability

The full dataset and replication code for the statistical analysis is available at the Open Science Framework https://osf.io/tr2e4/.
