# Supplementary material for: Resident funding and care home quality: a retrospective observational analysis of the impact of the two-tier care system in England
Source: Age Ageing. 2025 May 3;54(5):afaf100. doi: 10.1093/ageing/afaf100 (PMC12048780; doi:10.1093/ageing/afaf100)
Supplement: aa-24-1752-File002_afaf100 [file aa-24-1752-file002_afaf100.docx]

**Appendix for ‘Resident funding and care home quality: A retrospective observational analysis of the impact of the two-tier care system in England’**

Table of Contents

[Data 2](#_Toc188640483)

[Data coding procedure 2](#_Toc188640484)

[Removed observations 2](#_Toc188640485)

[Table A1: Replication of main results including all observations. 2](#_Toc188640486)

[Data quality and recoding 3](#_Toc188640487)

[Control variables 3](#_Toc188640488)

[Supplementary analyses 4](#_Toc188640489)

[Table A2: Replication of main results (Table 3 in manuscript), using ordered logistic regression on the 4-point CQC rating scale (Inadequate, Requires improvement, Good, Outstanding). 4](#_Toc188640490)

[Table A3: Full regression results of Table 3. 5](#_Toc188640491)

[Table A4: Replicating main results with only active care homes. 6](#_Toc188640492)

[Table A5: Replication of main results with only resident covariates 7](#_Toc188640493)

[Table A6: Interaction regression results – self-funding percentage interacted with ownership (column 1) area deprivation (IDAOPI) (column 2). 8](#_Toc188640494)

[Table A7: Effect of self-funding by care home ownership (for-profit, public, and third sector). 9](#_Toc188640495)

[Table A8: Replicating main results using IDAOPI vs IMD. 10](#_Toc188640496)

[Table A9: Percentage of self-funded residents by care home categories. 11](#_Toc188640497)

[Figure A1: Care home resident mix for care homes registered for old people and care homes not registered for older residents. 12](#_Toc188640498)

[Figure A2: Interaction effect – moderating effect of area deprivation on percentage of self-funded residents and quality. 13](#_Toc188640499)

[Sensitivity analysis 14](#_Toc188640500)

[Figure A3: Marginal effects of self-funding percentage on care quality (Good/Outstanding vs Inadequate/Requires improvement) by resident care needs. 14](#_Toc188640501)

[Figure A4: Three-way interaction between funding status, homes with at least one dementia resident, and ownership. 15](#_Toc188640502)

[Figure A5: Marginal effects of self-funding percentage on care quality (Good/Outstanding vs Inadequate/Requires improvement) by (a) homes registered for older resident and (b) homes with and without nursing. 16](#_Toc188640503)

[Figure A6: Predicted probabilities of care homes receiving an inadequate, requires improvement, good, or outstanding CQC inspection rating. 17](#_Toc188640504)

[Figure A7: Predicted probabilities of care homes receiving an inadequate, requires improvement, good, or outstanding CQC inspection rating - by ownership. 18](#_Toc188640505)

# Data

## Data coding procedure

The anonymised dataset specified whether each home had a companies house or charity number and its organisational form (individual, partnership, organisation, or NHS board). We used this information to code ownership: all care homes with a companies house number and individuals/partnerships without a charity number were coded for-profit. All homes with a charity number were coded third sector, and the rest were coded as public provision. The ownership proportions are equivalent to the publicly available CQC data.

The percentage of state- and self-funded residents were calculated relative to the total number of residents reported in the PIR data. If the reported number of self- or state-funded residents was 0 and the other category was not missing or 0, we coded the percentage to 100%.

## Removed observations

There were some observations for which the sum of self- and state-funded residents deviated slightly from the reported total number of residents, but often only by only a few residents. We removed all observations where the deviation was above 20% of the reported number of residents (n= 977). Below we replicate our main results using all observations; the exclusion of these observations did not change our results.

## Table A1: Replication of main results including all observations.

|  | (1) | (2) | (3) | (4) | (5) |
| --- | --- | --- | --- | --- | --- |
| Self-funders (%) | 1.003^***^ [1.002,1.004] | 1.002^**^ [1.001,1.003] | 1.010^***^ [1.009,1.012] | 1.010^***^ [1.008,1.012] | 1.012^***^ [1.010,1.014] |
| Ownership |  |  |  |  |  |
| (Reference:  for-profit) |  |  |  |  |  |
| Local authority | 2.238^***^ [1.592,3.147] |  |  | 2.265^***^ [1.600,3.205] | 2.094^***^ [1.439,3.049] |
| Third sector | 1.552^***^ [1.348,1.788] |  |  | 1.343^***^ [1.159,1.557] | 1.171 [0.976,1.404] |
| IDAOPI (deciles) |  | 1.022^*^ [1.005,1.039] |  | 1.009 [0.992,1.027] | 1.006 [0.986,1.027] |
| Older people |  |  | 0.762^***^ [0.664,0.875] | 0.765^***^ [0.665,0.880] |  |
| Nursing |  |  | 0.837^**^ [0.745,0.940] | 0.860^*^ [0.763,0.968] | 0.878^*^ [0.774,0.996] |
| Number of residents |  |  | 1.006^***^ [1.003,1.009] | 1.005^**^ [1.002,1.008] | 1.006^**^ [1.002,1.009] |
| Dementia needs (%) |  |  | 0.580^***^ [0.488,0.690] | 0.597^***^ [0.500,0.712] | 0.664^***^ [0.551,0.800] |
| MH needs (%) |  |  | 1.284^***^ [1.119,1.472] | 1.301^***^ [1.133,1.494] | 1.319^***^ [1.121,1.551] |
| Physical disabilities (%) |  |  | 1.074 [0.954,1.209] | 1.029 [0.913,1.160] | 1.016 [0.889,1.161] |
| Learning disabilities (%) |  |  | 1.445^***^ [1.223,1.707] | 1.367^***^ [1.152,1.622] | 1.777^***^ [1.425,2.217] |
| Staff-resident ratio |  |  | 1.008 [0.960,1.058] | 1.007 [0.960,1.056] | 1.036 [0.964,1.114] |
| Location status (active/closed) |  |  |  | 2.106^***^ [1.823,2.432] | 1.996^***^ [1.693,2.352] |
| *N* | 28459 | 28438 | 28006 | 27993 | 20626 |
| Fixed effects year | Yes | Yes | Yes | Yes | Yes |
| Care home registrations | All | All | All | All | Older residents |

Note: IDAOPI =1 refers to the most deprived and 10 = least deprived. Outcome is quality (‘Inadequate’/’Requires improvement’ vs ‘Good’/Outstanding’) All effects are in odds ratios; 95% confidence intervals in brackets. ^*^ *p* < 0.05, ^**^ *p* < 0.01, ^***^ *p* < 0.00

## Data quality and recoding

We received the PIR data in raw format. The raw dataset included 14948 unique care home IDs. We removed observations where the sum of reported self- and state-funded residents deviated with more than 20% from the reported total number of residents (n=977 observations, 504 care homes), providing us with a final sample of 14,444 unique care homes. There were very few missing observations in the variables we used from the PIR responses, and there were only 0.01% and 0.54% missing observations in the linked deprivation and inspection data, respectively. To control for staff-resident ratio, we divided the number of residents with the number of full-time equivalent staff. This ratio included some outlier values, which were removed from the analysis (n=440 observations, 362 care homes). Removing these observations did not influence the results (see Table A5 above).

## Control variables

In the full models, we adjust for the following control variables: number of residents, service user band registrations, whether the home includes nursing, and residents’ care needs (proportion of residents with dementia, mental health needs, learning disabilities/autism, and physical disabilities), and the full-time staff to resident ratio. The selected control variables account for key factors that may confound the relationship between care home quality and our main variables of interest.

The number of residents and service user band registrations control for the size and diversity of care needs within each facility, which can affect resource allocation and management practices. Nursing home status is included to differentiate between homes with varying levels of need. Residents' specific care needs (such as dementia, mental health issues, learning disabilities/autism, and physical disabilities) are controlled for as they impact the complexity and resources required for adequate care. The full-time staff to resident ratio is crucial in accounting for staffing levels, when estimating care quality. By including these variables, the analysis aims to isolate the effects of the funding source, care home ownership, and area deprivation on care home quality.

# Supplementary analyses

## **Table A2:** Replication of main results (Table 3 in manuscript), using ordered logistic regression on the 4-point CQC rating scale (Inadequate, Requires improvement, Good, Outstanding).

|  | (1) | (2) | (3) | (4) | (5) |
| --- | --- | --- | --- | --- | --- |
| Self-funders (%) | 1.005^***^ [1.004,1.006] | 1.004^***^ [1.003,1.005] | 1.013^***^ [1.011,1.014] | 1.012^***^ [1.010,1.014] | 1.014^***^ [1.012,1.016] |
| Ownership (reference: for-profit |  |  |  |  |  |
| LA | 1.683^***^ [1.350,2.098] |  |  | 1.675^***^ [1.336,2.101] | 1.700^***^ [1.298,2.226] |
| Third sector | 1.397^***^ [1.245,1.567] |  |  | 1.198^**^ [1.061,1.353] | 1.155 [0.980,1.360] |
| IDAOPI (decile) |  | 1.032^***^ [1.016,1.048] |  | 1.020^*^ [1.003,1.036] | 1.012 [0.993,1.032] |
| Older people |  |  | 0.741^***^ [0.657,0.836] | 0.741^***^ [0.656,0.837] |  |
| Nursing |  |  | 0.875^*^ [0.784,0.977] | 0.887^*^ [0.794,0.992] | 0.896 [0.795,1.010] |
| Number of residents |  |  | 1.008^***^ [1.005,1.012] | 1.008^***^ [1.004,1.011] | 1.008^***^ [1.005,1.012] |
| Dementia needs (%) |  |  | 0.995^***^ [0.994,0.997] | 0.995^***^ [0.994,0.997] | 0.997^***^ [0.995,0.999] |
| MH needs (%) |  |  | 1.002^***^ [1.001,1.004] | 1.003^***^ [1.001,1.004] | 1.003^***^ [1.001,1.005] |
| Physical disabilities (%) |  |  | 1.001 [0.999,1.002] | 1.000 [0.999,1.001] | 1.001 [0.999,1.002] |
| Learning disabilities (%) |  |  | 1.004^***^ [1.003,1.006] | 1.003^***^ [1.002,1.005] | 1.006^***^ [1.004,1.008] |
| Staff-resident ratio |  |  | 1.088^***^ [1.040,1.138] | 1.088^***^ [1.040,1.137] | 1.133^***^ [1.059,1.213] |
| Location status (active/closed) |  |  |  | 2.370^***^ [2.030,2.768] | 2.340^***^ [1.950,2.806] |
| *N* | 28316 | 28295 | 27873 | 27860 | 20599 |
| pseudo *R*^2^ | 0.005 | 0.003 | 0.017 | 0.023 | 0.026 |
| Fixed effects year | Yes | Yes | Yes | Yes | Yes |
| Care home registrations | All | All | All | All | Older residents |

Note: IDAOPI =1 refers to the most deprived and 10 = least deprived. Outcome is the 4-point scale (‘Inadequate’/’Requires improvement’ vs ‘Good’/Outstanding’) All effects are in odds ratios; 95% confidence intervals in brackets. ^*^ *p* < 0.05, ^**^ *p* < 0.01, ^***^ *p* < 0.001

## **Table A3:** Full regression results of Table 3.

|  | (1) | (2) | (3) | (4) | (5) |
| --- | --- | --- | --- | --- | --- |
| Self-funders (%) | 1.003^***^ [1.002,1.005] | 1.002^***^ [1.001,1.004] | 1.010^***^ [1.009,1.012] | 1.010^***^ [1.008,1.012] | 1.012^***^ [1.010,1.014] |
| Ownership |  |  |  |  |  |
| (Reference:  for-profit) |  |  |  |  |  |
| Local authority | 2.170^***^ [1.537,3.064] |  |  | 2.182^***^ [1.535,3.102] | 2.012^***^ [1.374,2.946] |
| Third sector | 1.556^***^ [1.349,1.794] |  |  | 1.348^***^ [1.161,1.563] | 1.181 [0.983,1.420] |
| IDAOPI (deciles) |  | 1.021^*^ [1.004,1.038] |  | 1.008 [0.991,1.026] | 1.006 [0.986,1.027] |
| Older people |  |  | 0.761^***^ [0.661,0.875] | 0.764^***^ [0.663,0.880] |  |
| Nursing |  |  | 0.839^**^ [0.747,0.943] | 0.861^*^ [0.764,0.969] | 0.876^*^ [0.772,0.994] |
| Number of residents |  |  | 1.006^***^ [1.003,1.009] | 1.005^**^ [1.002,1.008] | 1.006^**^ [1.002,1.009] |
| Dementia needs (%) |  |  | 0.994^***^ [0.993,0.996] | 0.995^***^ [0.993,0.997] | 0.996^***^ [0.994,0.998] |
| MH needs (%) |  |  | 1.002^***^ [1.001,1.004] | 1.002^***^ [1.001,1.004] | 1.003^**^ [1.001,1.004] |
| Physical disabilities (%) |  |  | 1.001 [0.999,1.002] | 1.000 [0.999,1.001] | 1.000 [0.999,1.002] |
| Learning disabilities (%) |  |  | 1.004^***^ [1.002,1.005] | 1.003^***^ [1.001,1.005] | 1.006^***^ [1.003,1.008] |
| Staff-resident ratio |  |  | 1.016 [0.968,1.067] | 1.016 [0.968,1.066] | 1.043 [0.969,1.122] |
| Location status (active/closed) |  |  |  | 2.126^***^ [1.841,2.455] | 2.037^***^ [1.730,2.399] |
| *N* | 28316 | 28295 | 27873 | 27860 | 20599 |
| Unique location clusters | 14,300 | 14,287 | 14,182 | 14,177 | 10,532 |
| Fixed effects year | Yes | Yes | Yes | Yes | Yes |
| Care home registrations | All | All | All | All | Older residents |

Note: IDAOPI =1 refers to the most deprived and 10 = least deprived. Outcome is quality (‘Inadequate’/’Requires improvement’ vs ‘Good’/Outstanding’) All effects are in odds ratios; 95% confidence intervals in brackets. ^*^ *p* < 0.05, ^**^ *p* < 0.01, ^***^ *p* < 0.001.

## **Table A4:** Replicating main results with only active care homes.

|  | (1) | (2) |
| --- | --- | --- |
| Self-funders (%) | 1.011^***^ [1.008,1.013] | 1.012^***^ [1.010,1.015] |
| Ownership |  |  |
| (Reference:  for-profit) |  |  |
| Local authority | 2.097^***^ [1.445,3.045] | 1.907^**^ [1.270,2.863] |
| Third sector | 1.418^***^ [1.204,1.669] | 1.250^*^ [1.025,1.525] |
| Older people | 0.751^***^ [0.647,0.872] |  |
| Nursing | 0.852^*^ [0.752,0.965] | 0.873^*^ [0.764,0.996] |
| IDAOPI (deciles) | 1.007 [0.988,1.026] | 1.003 [0.982,1.025] |
| Number of residents | 1.005^**^ [1.001,1.008] | 1.005^**^ [1.002,1.009] |
| Dementia needs (%) | 0.995^***^ [0.993,0.997] | 0.996^***^ [0.995,0.998] |
| MH needs (%) | 1.002^**^ [1.001,1.004] | 1.003^**^ [1.001,1.004] |
| Physical disabilities (%) | 1.000 [0.999,1.002] | 1.000 [0.999,1.002] |
| Learning disabilities (%) | 1.003^**^ [1.001,1.005] | 1.006^***^ [1.003,1.008] |
| Staff-resident ratio | 1.041 [0.988,1.097] | 1.052 [0.974,1.137] |
| *N* | 26315 | 19472 |
| Unique location clusters | 13,027 | 9,671 |
| Fixed effects year | Yes | Yes |
| Care home registrations | All | Older residents |
| Registration status | Active | Active |

Note: IDAOPI =1 refers to the most deprived and 10 = least deprived. Outcome is quality (‘Inadequate’/’Requires improvement’ vs ‘Good’/Outstanding’) All effects are in odds ratios; 95% confidence intervals in brackets. ^*^ *p* < 0.05, ^**^ *p* < 0.01, ^***^ *p* < 0.001

## Table A5: Replication of main results with only resident covariates

|  | (1) | (2) |
| --- | --- | --- |
| Self-funders (%) | 1.010^***^ [1.008,1.012] | 1.012^***^ [1.010,1.014] |
| Ownership |  |  |
| (Reference:  for-profit) |  |  |
| Local authority | 2.302^***^ [1.616,3.280] | 2.179^***^ [1.484,3.198] |
| Third sector | 1.328^***^ [1.146,1.540] | 1.181 [0.983,1.419] |
| Older people | 0.757^***^ [0.658,0.872] |  |
| Nursing | 0.864^*^ [0.769,0.971] | 0.889 [0.787,1.005] |
| IDAOPI (deciles) | 1.009 [0.991,1.027] | 1.006 [0.986,1.026] |
| Number of residents | 1.005^**^ [1.001,1.008] | 1.005^**^ [1.002,1.009] |
| Dementia needs (%) | 0.995^***^ [0.993,0.996] | 0.996^***^ [0.994,0.997] |
| MH needs (%) | 1.002^***^ [1.001,1.004] | 1.003^**^ [1.001,1.004] |
| Physical disabilities (%) | 1.000 [0.999,1.001] | 1.000 [0.999,1.001] |
| Learning disabilities (%) | 1.003^***^ [1.001,1.005] | 1.006^***^ [1.004,1.008] |
| Location status (active/closed) | 2.121^***^ [1.839,2.446] | 2.018^***^ [1.716,2.373] |
| *N* | 28280 | 20847 |
| Unique location clusters | 14,279 | 10,588 |
| Fixed effects year | Yes | Yes |
| Care home registrations | All | Older residents |

Note: IDAOPI =1 refers to the most deprived and 10 = least deprived. Outcome is quality (‘Inadequate’/’Requires improvement’ vs ‘Good’/Outstanding’) All effects are in odds ratios; 95% confidence intervals in brackets. ^*^ *p* < 0.05, ^**^ *p* < 0.01, ^***^ *p* < 0.001

## **Table A6:** Interaction results – self-funding percentage interacted with ownership (column 1) area deprivation (IDAOPI) (column 2).

|  | (1) | (2) |
| --- | --- | --- |
| Self-funders (%) | 1.011^***^ [1.009,1.013] | 1.004 [1.000,1.008] |
| Ownership |  |  |
| (Reference:  for-profit) |  |  |
| Local authority | 2.704^***^ [1.861,3.930] | 2.166^***^ [1.525,3.078] |
| Third sector | 1.515^***^ [1.260,1.821] | 1.357^***^ [1.169,1.575] |
| **Interaction**: LA * self-funders (%) | 0.984^***^ [0.974,0.993] |  |
| **Interaction:** Third sector * self-funders (%) | 0.995^*^ [0.991,0.999] |  |
| **Interaction:** IDAOPI & self-funders (%) |  | 1.001^***^ [1.000,1.002] |
| IDAOPI (deciles) | 1.007 [0.989,1.025] | 0.985 [0.964,1.007] |
| Older people | 0.767^***^ [0.666,0.884] | 0.765^***^ [0.664,0.882] |
| Nursing | 0.860^*^ [0.763,0.969] | 0.856^*^ [0.760,0.964] |
| Number of residents | 1.005^**^ [1.002,1.008] | 1.005^**^ [1.002,1.008] |
| Dementia needs (%) | 0.995^***^ [0.993,0.996] | 0.995^***^ [0.993,0.997] |
| MH needs (%) | 1.003^***^ [1.001,1.004] | 1.002^**^ [1.001,1.004] |
| Physical disabilities (%) | 1.000 [0.999,1.001] | 1.000 [0.999,1.002] |
| Learning disabilities (%) | 1.003^***^ [1.001,1.005] | 1.003^***^ [1.001,1.005] |
| Staff-resident ratio | 1.019 [0.971,1.070] | 1.017 [0.969,1.068] |
| Location status (active/closed) | 2.135^***^ [1.848,2.466] | 2.125^***^ [1.840,2.454] |
| *N* | 27860 | 27860 |
| Unique location clusters | 14,177 | 14,177 |
| Fixed effects year | Yes | Yes |
| Care home registrations | All | All |

Model 1 is used to estimate Figure 2 in the Manuscript. Note: IDAOPI =1 refers to the most deprived and 10 = least deprived areas. Outcome is quality (‘Inadequate’/’Requires improvement’ vs ‘Good’/Outstanding’) All effects are in odds ratios; 95% confidence intervals in brackets. ^*^ *p* < 0.05, ^**^ *p* < 0.01, ^***^ *p* < 0.001

## **Table A7:** Effect of self-funding by care home ownership (for-profit, public, and third sector).

|  | (3) | (4) | (5) |
| --- | --- | --- | --- |
| Self-funders (%) | 1.011^***^ [1.009,1.013] | 0.999 [0.987,1.012] | 1.003 [0.997,1.009] |
| IDAOPI (deciles) | 1.011 [0.992,1.031] | 0.884^*^ [0.785,0.996] | 0.993 [0.941,1.047] |
| Older people | 0.805^**^ [0.688,0.941] | 0.725 [0.203,2.590] | 0.565^**^ [0.398,0.802] |
| Nursing | 0.888 [0.784,1.006] | 0.755 [0.263,2.169] | 0.592^*^ [0.390,0.898] |
| Number of residents | 1.005^**^ [1.001,1.008] | 0.992 [0.971,1.013] | 1.005 [0.995,1.015] |
| Dementia needs (%) | 0.995^***^ [0.993,0.997] | 0.984^**^ [0.973,0.996] | 0.997 [0.990,1.003] |
| MH needs (%) | 1.003^***^ [1.001,1.004] | 1.002 [0.992,1.011] | 1.002 [0.997,1.006] |
| Physical disabilities (%) | 1.000 [0.999,1.001] | 0.997 [0.988,1.006] | 1.002 [0.998,1.005] |
| Learning disabilities (%) | 1.003^***^ [1.001,1.005] | 1.003 [0.991,1.016] | 1.000 [0.995,1.005] |
| Staff-resident ratio | 1.036 [0.983,1.093] | 1.073 [0.666,1.727] | 0.897 [0.782,1.030] |
| Location status (active/closed) | 2.011^***^ [1.716,2.356] | 1.408 [0.487,4.072] | 3.092^***^ [2.136,4.475] |
| *N* | 23609 | 656 | 3595 |
| Unique location clusters | 12,004 | 351 | 1,822 |
| Fixed effects year | Yes | Yes | Yes |
| Ownership | For-profit | Public | Non-profit |

Note: IDAOPI =1 refers to the most deprived and 10 = least deprived. Outcome is quality (‘Inadequate’/’Requires improvement’ vs ‘Good’/Outstanding’) All effects are in odds ratios; 95% confidence intervals in brackets. ^*^ *p* < 0.05, ^**^ *p* < 0.01, ^***^ *p* < 0.001

## **Table A8:** Replicating main results using IDAOPI vs IMD.

|  | (1) | (2) |
| --- | --- | --- |
| Self-funders (%) | 1.010^***^ [1.008,1.012] | 1.010^***^ [1.008,1.012] |
| Ownership |  |  |
| (Reference:  for-profit) |  |  |
| Local authority | 2.182^***^ [1.535,3.102] | 2.181^***^ [1.535,3.099] |
| Third sector | 1.348^***^ [1.161,1.563] | 1.345^***^ [1.159,1.561] |
| IDAOPI (deciles) | 1.008 [0.991,1.026] |  |
| IMD (deciles) |  | 1.009 [0.992,1.026] |
| Older people | 0.764^***^ [0.663,0.880] | 0.764^***^ [0.663,0.881] |
| Nursing | 0.861^*^ [0.764,0.969] | 0.860^*^ [0.764,0.969] |
| Number of residents | 1.005^**^ [1.002,1.008] | 1.005^**^ [1.002,1.008] |
| Dementia needs (%) | 0.995^***^ [0.993,0.997] | 0.995^***^ [0.993,0.997] |
| MH needs (%) | 1.002^***^ [1.001,1.004] | 1.002^***^ [1.001,1.004] |
| Physical disabilities (%) | 1.000 [0.999,1.001] | 1.000 [0.999,1.001] |
| Learning disabilities (%) | 1.003^***^ [1.001,1.005] | 1.003^***^ [1.001,1.005] |
| Staff-resident ratio | 1.016 [0.968,1.066] | 1.016 [0.968,1.067] |
| Location status (active/closed) | 2.126^***^ [1.841,2.455] | 2.126^***^ [1.841,2.455] |
| *N* | 27860 | 27860 |
| Unique location clusters | 14,177 | 14,177 |
| Fixed effects year | Yes | Yes |
| Care home registrations | All | All |

Note: IDAOPI =1 refers to the most deprived and 10 = least deprived. Outcome is quality (‘Inadequate’/’Requires improvement’ vs ‘Good’/Outstanding’) All effects are in odds ratios; 95% confidence intervals in brackets. ^*^ *p* < 0.05, ^**^ *p* < 0.01, ^***^ *p* < 0.001

## **Table A9:** Percentage of state- and self-funded residents by care home categories. The counts and percentages are based on residents in active homes as of September 2023.

|  | Self-funded residents % (n) | State-funded residents % (n) | Total |
| --- | --- | --- | --- |
| Care home ownership | |  |  |
| For-profit | 35.7% (106,964) | 64.3% (192,260) | 299,224 |
| Local Authority (public) | 13.6% (1,275) | 86.4% (8,099) | 9,374 |
| Third sector (non-profit) | 39.5% (13,412) | 60.5% (20,553) | 33,965 |
| Area deprivation (IDAOPI deciles) | | |  |
| 1 (most deprived) | 16.2% (3,707) | 83.8% (19,071) | 22,778 |
| 2 | 23.4% (7,569) | 76.6% (24,740) | 32,309 |
| 3 | 25.8% (9,627) | 74.2% (27,623) | 37,250 |
| 4 | 29.9% (11,591) | 70.1% (27,144) | 38,735 |
| 5 | 35.7% (14,264) | 64.3% (25,742) | 40,006 |
| 6 | 37.5% (15,269) | 62.5% (25,470) | 40,739 |
| 7 | 39.4% (14,763) | 60.6% (22,733) | 37,496 |
| 8 | 45.3% (15,769) | 54.7% (19,060) | 34,829 |
| 9 | 47.4% (15,811) | 52.6% (17,551) | 33,362 |
| 10 (least deprived) | 53.4% (13,215) | 46.6% (11,518) | 24,733 |
| Overall CQC ratings | |  |  |
| Inadequate | 27.2% (1,378) | 72.8% (3,674) | 5,052 |
| Requires improvement | 28.8% (18,208) | 71.2% (45,140) | 63,348 |
| Good | 36.3% (92,637) | 63.7% (162,591) | 255,228 |
| Outstanding | 50.5% (8,373) | 49.5% (8,186) | 16,559 |
| Number of residents | | |  |
| 1-9 | 3.5% (704) | 96.5% (19,615) | 20,319 |
| 10-19 | 26.9% (9,234) | 73.1% (25,109) | 34,343 |
| 20-49 | 37.9% (65,477) | 62.1% (107,259) | 172,736 |
| Over 50 | 40.1% (44,204) | 59.9% (65,917) | 110,121 |
| Number of staff | | |  |
| 1-9 | 14.8% (1,584) | 85.2% (9,092) | 10,676 |
| 10-19 | 21.8% (8,147) | 78.2% (29,202) | 37,349 |
| 20-49 | 36.1% (52,447) | 63.9% (92,745) | 145,192 |
| Over 50 | 40.0% (57,840) | 60.0% (86,836) | 144,676 |
| Care home registrations | | |  |
| Older residents | | |  |
| No older residents | 5.2% (1,678) | 94.8% (30,470) | 32,148 |
| Older residents | 38.6% (119,973) | 61.4% (190,442) | 310,415 |
| Nursing |  |  |  |
| With nursing | 35.5% (64,385) | 64.5% (116,917) | 181,302 |
| Without nursing | 35.6% (57,258) | 64.4% (103,719) | 160,977 |
| Residents’ care needs |  |  |  |
| In a home with at least one resident with dementia needs | | |  |
| No | 5.7% (2,170) | 94.3% (35,748) | 37,918 |
| Yes | 39.2% (119,481) | 60.8% (185,164) | 304,645 |
| In a home with at least one resident with a physical disability | | |  |
| No | 32.6% (22,753) | 67.4% (47,049) | 69,802 |
| Yes | 36.3% (98,898) | 63.7% (173,863) | 272,761 |
| In a home with at least one resident with mental health needs | | | |
| No | 36.0% (111,107) | 64.0% (197,616) | 308,723 |
| Yes | 31.2% (10,544) | 68.8% (23,296) | 33,840 |
| In a home with at least one resident with a learning disability or autism | | | |
| No | 45.8% (85,827) | 54.2% (101,611) | 187,438 |
| Yes | 23.1% (35,824) | 76.9% (119,301) | 155,125 |

## **Figure A1:** Care home resident mix for care homes registered for old people and care homes not registered for older residents.

**
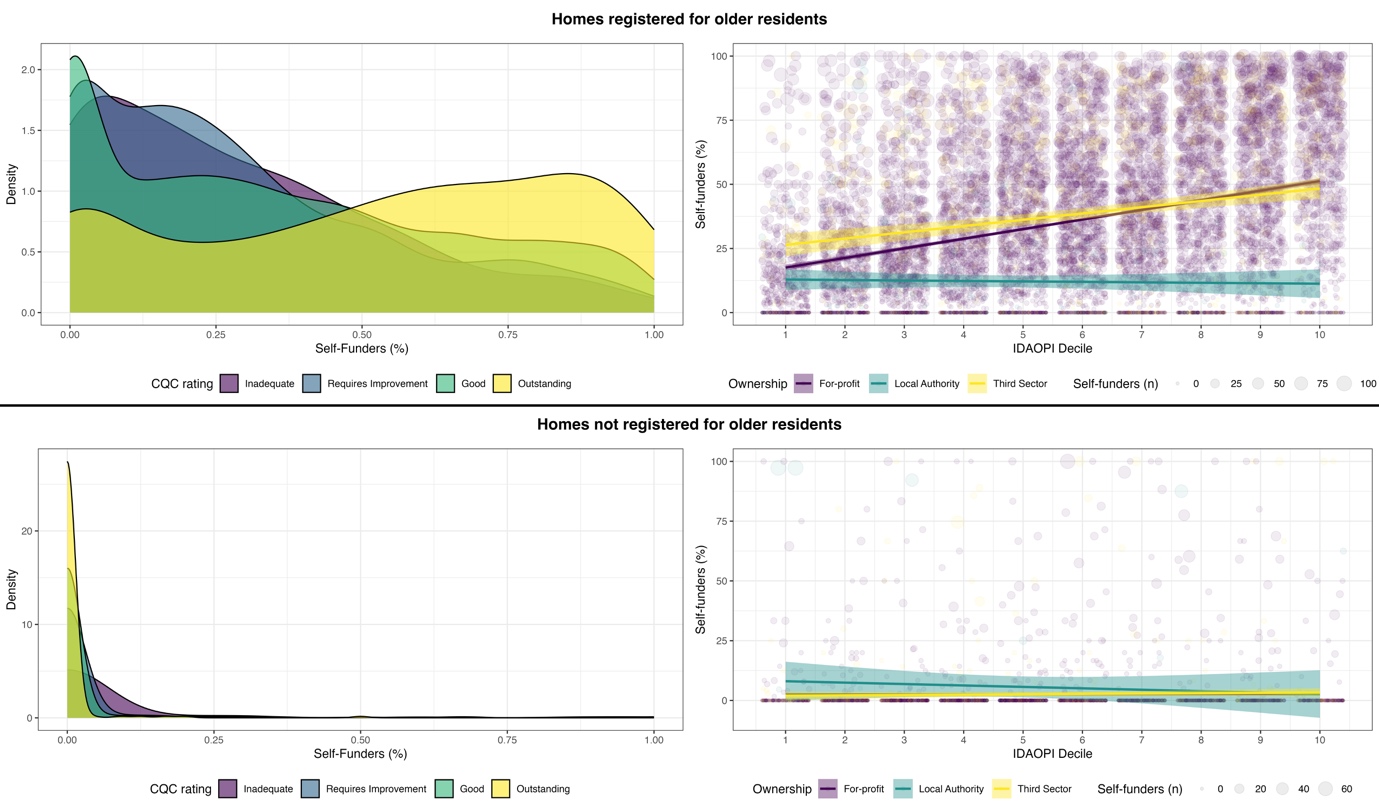
**

## **Figure A2:** Interaction effect – moderating effect of area deprivation on percentage of self-funded residents and quality.

**Note:** IDAOPI =1 refers to the most deprived and 10 = least deprived.

# Sensitivity analysis

Figures A3-A5 below show that the quality of homes without older residents does not vary according to funding status (Figure A3, panel (A)), which is consistent with there being very few self-funded residents in such homes (Table 2). To test if this influences our main results, we ran a three-way interaction between ownership, funding status, and homes with/without dementia residents. The model (Figures A4-5) shows that the link between quality and the percentage of self-funders is strongest for for-profit homes with older and dementia residents.

Figure A3: Marginal effects of self-funding percentage on care quality (Good/Outstanding vs Inadequate/Requires improvement) by resident care needs.

All models are adjust for the variables in Model 3, Table A3.

Figure A4: Three-way interaction between funding status, homes with at least one dementia resident, and ownership.

All models are adjusted for the variables in Model 3, Table A3. Panel (a) shows the marginal effects of self-funding percentage on care quality (Good/Outstanding vs Inadequate/Requires improvement) by ownership and dementia needs. Panel (b) plots the differences in predicted probabilities by ownership and homes with and without dementia residents.

Figure A5: Marginal effects of self-funding percentage on care quality (Good/Outstanding vs Inadequate/Requires improvement) by (a) homes registered for older resident and (b) homes with and without nursing.

All models are adjusted for the variables in Model 3, Table A3.

Figure A6: Predicted probabilities of care homes receiving an inadequate, requires improvement, good, or outstanding CQC inspection rating.

**Note:** The predicted probabilities are calculated using the ordered logistic regression results from model 3 in Table A2.

Figure A7: Predicted probabilities of care homes receiving an inadequate, requires improvement, good, or outstanding CQC inspection rating - by ownership.

**Note**: The predicted probabilities are calculated using the ordered logistic regression results from model 3 in Table A2. The models include an interaction term for ownership and self-funding percentage.
